# Interaction of the Human Papillomavirus E6 Oncoprotein with Sorting Nexin 27 Modulates Endocytic Cargo Transport Pathways

**DOI:** 10.1371/journal.ppat.1005854

**Published:** 2016-09-20

**Authors:** Ketaki Ganti, Paola Massimi, Joaquin Manzo-Merino, Vjekoslav Tomaić, David Pim, Martin P. Playford, Marcela Lizano, Sally Roberts, Christian Kranjec, John Doorbar, Lawrence Banks

**Affiliations:** 1 International Centre for Genetic Engineering and Biotechnology, Padriciano, Trieste, Italy; 2 CONACyT Research Fellow, Instituto Nacional de Cancerologia, Mexico/Instituto de Investigaciones Biomedicas, Universidad Nacional Autonoma de Mexico. Col. Seccion XVI, Tlalpan, Mexico; 3 Division of Molecular Medicine, Ruđjer Bošković Institute, Zagreb, Croatia; 4 National Heart, Blood and Lung Institute, NIH, Bethesda, Maryland, United States of America; 5 Instituto Nacional de Cancerologia, Mexico/Instituto de Investigaciones Biomedicas, Universidad Nacional Autonoma de Mexico. Col. Seccion XVI, Tlalpan, Mexico; 6 Institute of Cancer and Genomic Sciences, University of Birmingham, Birmingham, United Kingdom; 7 Department of Pathology, University of Cambridge, Tennis Court Road, Cambridge, United Kingdom; University of Wisconsin Madison School of Medicine and Public Health, UNITED STATES

## Abstract

A subset of high-risk Human Papillomaviruses (HPVs) are the causative agents of a large number of human cancers, of which cervical is the most common. Two viral oncoproteins, E6 and E7, contribute directly towards the development and maintenance of malignancy. A characteristic feature of the E6 oncoproteins from cancer-causing HPV types is the presence of a PDZ binding motif (PBM) at its C-terminus, which confers interaction with cellular proteins harbouring PDZ domains. Here we show that this motif allows E6 interaction with Sorting Nexin 27 (SNX27), an essential component of endosomal recycling pathways. This interaction is highly conserved across E6 proteins from multiple high-risk HPV types and is mediated by a classical PBM-PDZ interaction but unlike many E6 targets, SNX27 is not targeted for degradation by E6. Rather, in HPV-18 positive cell lines the association of SNX27 with components of the retromer complex and the endocytic transport machinery is altered in an E6 PBM-dependent manner. Analysis of a SNX27 cargo, the glucose transporter GLUT1, reveals an E6-dependent maintenance of GLUT1 expression and alteration in its association with components of the endocytic transport machinery. Furthermore, knockdown of E6 in HPV-18 positive cervical cancer cells phenocopies the loss of SNX27, both in terms of GLUT1 expression levels and its vesicular localization, with a concomitant marked reduction in glucose uptake, whilst loss of SNX27 results in slower cell proliferation in low nutrient conditions. These results demonstrate that E6 interaction with SNX27 can alter the recycling of cargo molecules, one consequence of which is modulation of nutrient availability in HPV transformed tumour cells.

## Author Summary

A unique feature of the high risk Human Papillomavirus (HPV) E6 oncoproteins is the presence of a PDZ binding motif (PBM) on its extreme C-terminus. This motif confers on E6 an ability to interact with a number of cellular proteins which possess PDZ domains, and this activity of E6 is important during the viral life cycle and contributes towards HPV-induced malignancy. In this study we describe a novel activity of high risk HPV E6 oncoproteins involving the direct regulation of endocytic transport pathways. This activity is dependent upon the E6 PBM and involves interaction with the endocytic cargo sorting machinery via sorting nexin 27 (SNX27). One of the consequences of this interaction is a redistribution of SNX27 with respect to components of the retromer complex and this in turn affects the composition of the endocytic transport machinery. This impacts directly upon rates of cargo recycling and in the case of HPV transformed cells, contributes towards maintaining high levels of glucose uptake. This study therefore describes a new function for the E6 oncoproteins and sheds light on how HPVs can modulate endocytic transport pathways.

## Introduction

Human Papillomaviruses (HPVs) are the causative agents of a large number of human malignancies, chief among which is cervical cancer, with over 500,000 reported cases worldwide annually [1,2]. There are currently more than 150 known types of HPVs, but not all of them are etiological agents of carcinomas. The cancer-causing HPVs are classified as “high-risk” types and these include HPV-16 and HPV-18, among others [3]. A hallmark of HPV induced-malignancy is the continued expression of the viral oncoproteins E6 and E7 throughout the course of tumour development [4,5]. Inhibiting the expression of either oncoprotein in cells derived from cervical tumours results in cell growth arrest and induction of apoptosis, demonstrating a continued requirement for E6 and E7 in the maintenance of the transformed phenotype [6]. Both viral oncoproteins act cooperatively, where E7 reprograms the infected cell to enter S phase by targeting, in part, the pRb family members, thus allowing the E2F family of transcription factors to transactivate various cell cycle genes [7–9]. The E6 oncoprotein complements the action of E7 by curbing the cell’s pro-apoptotic response to unscheduled DNA replication and targets pro-apoptotic proteins such as p53 [10] and Bak [11] for proteasome-mediated degradation via the action of the E6AP ubiquitin ligase [12]. However the ability of both E6 and E7 to contribute to cancer development depends upon a large number of other important interactions. In the case of the high-risk E6 oncoproteins a typical example is interaction with cellular PDZ (PSD-95/DLG/ZO-1) domain containing proteins.

A unique characteristic of the cancer-causing E6 oncoproteins is the presence of a PDZ binding motif (PBM) on their carboxy termini [13]. An intact E6 PBM is important for the ability of E6 to cooperate with E7 in the generation of tumours in transgenic mouse models, and also has transforming potential in some tissue culture models [14–16]. In the context of the whole viral genome, loss of E6 PBM function results in a defective replicative life cycle, with reduced levels of viral DNA amplification and, ultimately, loss of the viral episomes [17,18]. A large number of cellular PDZ domain-containing targets of E6 have been reported, with some of the best-characterised being a group of proteins involved in the regulation of cell polarity [19]. These include the Discs Large and Scribble proteins, which are key regulators of cell polarity and potential tumour suppressor proteins [20,21]. In addition, MAGI-1 would also appear to be a relevant target of E6, affecting the integrity of tight junctions in HPV-positive cells [22]. Recent high throughput screens have identified many further potential PDZ domain-containing targets of E6, with Sorting Nexin 27 (SNX27) being one such intriguing candidate [23,24].

A critical element in epithelial organisation is the regulation of polarity, and whilst key elements of the pathway, such as Scrib and Dlg, are well defined, it is clear that endocytic transport pathways also play important roles in epithelial organisation and polarity control [25–27]. Within these pathways, the retromer plays an important part in the transport of proteins from endosomes to the trans-Golgi network [28]. The retromer is an oligomeric protein complex formed by a Vacuolar protein sorting (Vps) subcomplex, consisting of Vps26, Vps29 and Vps35, and a heterodimer of sorting nexins (SNXs). SNXs are proteins characterized by the presence of a phosphoinositide binding PX domain, which targets them to phosphatidylinositol-3-monophosphate-rich membranes of the endosomes [29]. Many members of the SNX family also contain a number of protein-protein interaction domains, through which they interact with their respective cargoes and ensure appropriate trafficking [29–32]. Of particular interest from the HPV E6 point of view is SNX27. It contains a C-terminal Ras Association/FERM like domain, which has been shown to associate with the Ras GTPase [33], and SNX27 is the only SNX that contains a PDZ domain [30,32,34]. Recent proteomic studies have indicated that HPV-16 and HPV-18 E6 are potential interacting partners of SNX27 [23,24]. The SNX27 PDZ domain mediates the interaction with various PBM-containing cargoes and is thought to regulate the trafficking of these proteins through the endosomal pathway [35–38]. SNX27-mediated recycling of PBM-containing cargoes from endosomes to the plasma membrane is dependent on its interaction with the retromer, and recent studies have shown that the SNX27-retromer link is crucial for the retrieval and recycling of various transmembrane proteins. These include proteins required for maintenance of cellular homeostasis and growth, among which is GLUT-1, which is essential for glucose uptake [39,40]. We therefore initiated a series of studies to investigate whether E6 can associate with SNX27 and, if so, to ask what are the potential implications of this interaction in the recycling function of the SNX27-retromer complex in the context of GLUT-1 as a cargo.

## Results

### Sorting Nexin 27 interacts with multiple high-risk HPV E6 types in a PBM-dependent manner

**Fig 1:**
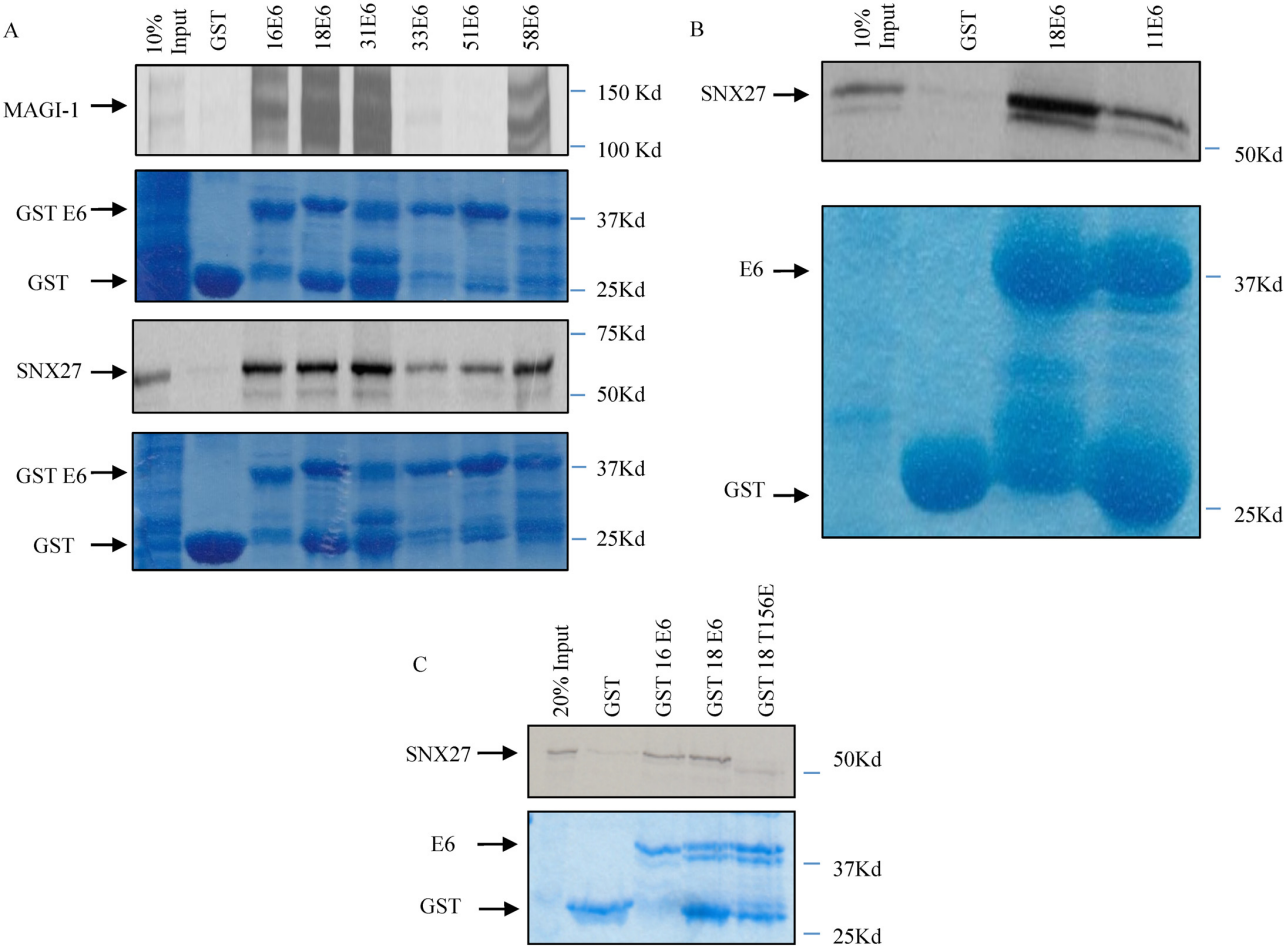
SNX27 interacts with multiple high-risk HPV E6 proteins. Panel A: SNX27 and MAGI-1 were translated and radio-labelled *in vitro* using the TNT Coupled Reticulocyte Lysate System (Promega). The translated proteins were then incubated with purified GST-tagged HPV E6s as indicated. The arrows indicate the position of MAGI-1 and SNX27 on the autoradiograms, which show the levels of bound and input proteins. The Coomassie Blue staining shows loading of the purified GST proteins, with the arrows indicating the position of GST and the GST tagged E6s. Panel B: SNX27 was translated and radio-labelled *in vitro* using the TNT Coupled Reticulocyte Lysate System (Promega). The translated proteins were then incubated with purified GST alone, GST-HPV18 E6 or GST-HPV11 E6 as indicated, with the upper panel showing the autoradiogram of bound and input SNX27. The Coomassie Blue staining shows loading of the purified GST proteins. Arrows indicate the position of GST and the GST-tagged E6s. Panel C: SNX27 was translated and radio-labelled *in vitro* using the TNT Coupled Reticulocyte Lysate System (Promega). The translated proteins were then incubated with purified GST alone, GST-HPV16 E6, GST-HPV18 E6 or the GST-HPV18 E6 T156E mutant as indicated. The upper panel shows the autoradiogram of bound and input proteins with the arrows indicating the position of SNX27. The Coomassie Blue staining shows loading of the purified GST proteins, with the arrows indicating the position of GST and the GST-tagged E6s.

Proteomic screens for cellular interacting partners of HPV E6 identified the PDZ domain containing protein SNX27 as a potential novel target of the high risk HPV E6 proteins [23,24]. To verify if this is indeed a *bona-fide* interaction, a series of in vitro binding assays were performed. The E6 proteins derived from the high-risk HPV-16, 18, 31, 33, 51 and 58 were expressed as GST fusion proteins and purified. These were then incubated with in vitro translated and radiolabelled SNX27 for 2 hours at 4°C. MAGI-1, which has been shown previously to be a strongly bound PDZ domain-containing target of HPV-16 and HPV-18 E6 was used as a positive control [41]. After thorough washing of the beads, the level of binding was determined by SDS-PAGE and autoradiography. The results in Fig 1A show that SNX27 is indeed a strong interaction partner of all the high-risk E6 types tested.

The E6 proteins from low-risk HPV types lack PBMs, therefore we wished to determine whether the ability of E6 to interact with SNX27 was restricted to high-risk types. To do this, pull-down assays were performed using purified GST-HPV18 E6 and GST-HPV11 E6 fusion proteins with in vitro translated and radiolabeled SNX27. The results in Fig 1B demonstrate very strong association between HPV-18 E6 and SNX27, with much weaker, albeit still detectable, association with HPV-11 E6. To determine whether the interaction between the high-risk E6 proteins and SNX27 required the E6 PBM, the assay was repeated using wild type HPV-18 E6 and the HPV-18 E6 T156E mutation, which has been shown previously to abolish PDZ recognition [42]. The results in Fig 1C demonstrate that the T156E mutation dramatically reduces the ability of E6 to interact with SNX27, although some residual interaction is still detectable. Taken together these results demonstrate that HPV-18 E6 interacts with SNX27 primarily through its PBM, although other residues within E6 are also most likely to be involved, and this is reflected in a weak degree of association between HPV-11 E6 and SNX27.

**Fig 2:**
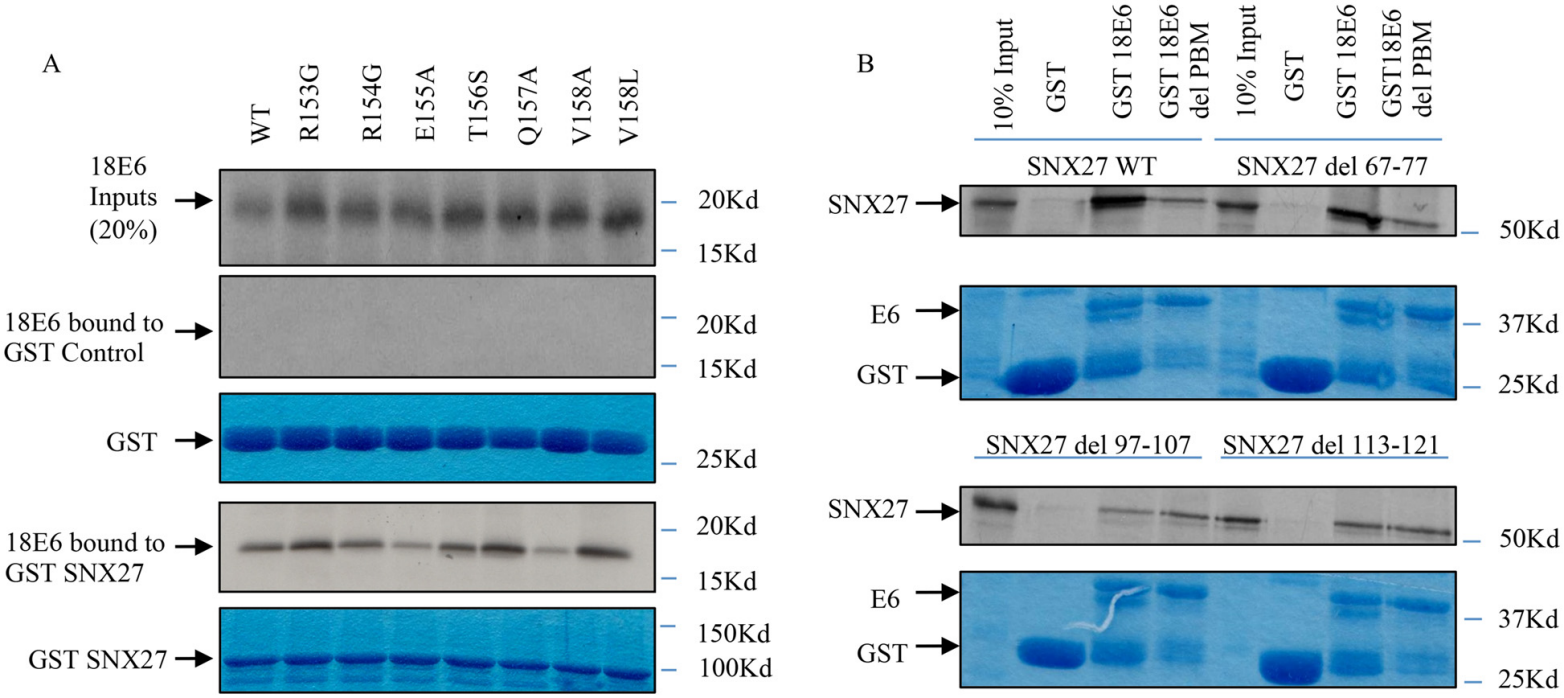
Interaction between HPV-18 E6 and SNX27 involves PBM-PDZ recognition. Panel A: The C-terminal HPV 18E6 mutants were translated and radio-labelled *in vitro* using the TNT Coupled Reticulocyte Lysate System (Promega). The translated proteins were then incubated with purified GST alone or GST-tagged SNX27 as indicated. The top panel shows input levels of the different HPV E6 mutants. The lower pairs of panels show the Coomassie stained gels of input GST and GST-SNX27 (lower panel in each pair) and the autoradiograms of the bound E6 (upper panel in each pair). Panel B: Wild type SNX27 and the various SNX27 deletion mutants was translated *in vitro* in rabbit reticulocyte lysate and radio labelled using the TNT Coupled Reticulocyte Lysate System (Promega). The translated proteins were then incubated with purified GST alone, GST-HPV18 E6 or the GST-HPV18 E6ΔPBM mutant as indicated. The arrows indicate the position of SNX27 on the autoradiograms, showing the levels of input and bound proteins. The Coomaasie Blue staining shows loading of the purified GST proteins.

Since SNX27 is a PDZ domain-containing protein, we wanted to determine if the interaction between SNX27 and E6 is through PBM-PDZ recognition. To do this, various C-terminal mutants of HPV-18 E6 [42] were translated and radiolabelled in vitro and used in pull down assays with purified GST-SNX27. The results in Fig 2A show that the mutation V158A, which destroys an essential part of the HPV-18 E6 PBM [42], greatly reduces the interaction with SNX27. Furthermore, the E155A mutation also severely compromises E6 recognition of SNX27, whilst the other mutants had either minimal effects or increased the level of association. This differential contribution of specific residues within the E6 PBM is in agreement with how E6 recognises other PDZ domain-containing substrates [43]. Taken together, these results demonstrate that HPV-18 E6 recognises SNX27 via sequences within the E6 PBM although, in agreement with the T156E mutation, other residues are also likely to be involved.

To confirm that the PDZ domain of SNX27 is necessary for E6 binding, PDZ deletion mutants of SNX27 were used in a series of pull down assays with GST-HPV18 E6 fusion proteins. As can be seen from Fig 2B, wild type SNX27 binds to HPV-18 E6 very strongly, but this interaction is greatly reduced with the HPV-18 E6ΔPBM mutant. The deletion of the retromer binding region of SNX27, spanning amino acids 67–77 [44] in the outer extended loop of the SNX27 PDZ domain, does not affect the interaction with HPV-18 E6. Interestingly, deletion of amino acids 97–107 and 113–121 in the core of the SNX27 PDZ domain markedly reduces the interaction with wild type HPV-18 E6, but does not abolish it. When the SNX27 mutants are used in binding assays with the E6ΔPBM mutant, interaction is found at a level similar to that seen with the wild type HPV-18 E6. These results demonstrate that the major site of recognition between HPV-18 E6 and SNX27 involves a classic PBM-PDZ interaction, although a second weaker interaction site also exists.

### SNX27 distribution is altered by E6 in a PBM dependent manner

**Fig 3:**
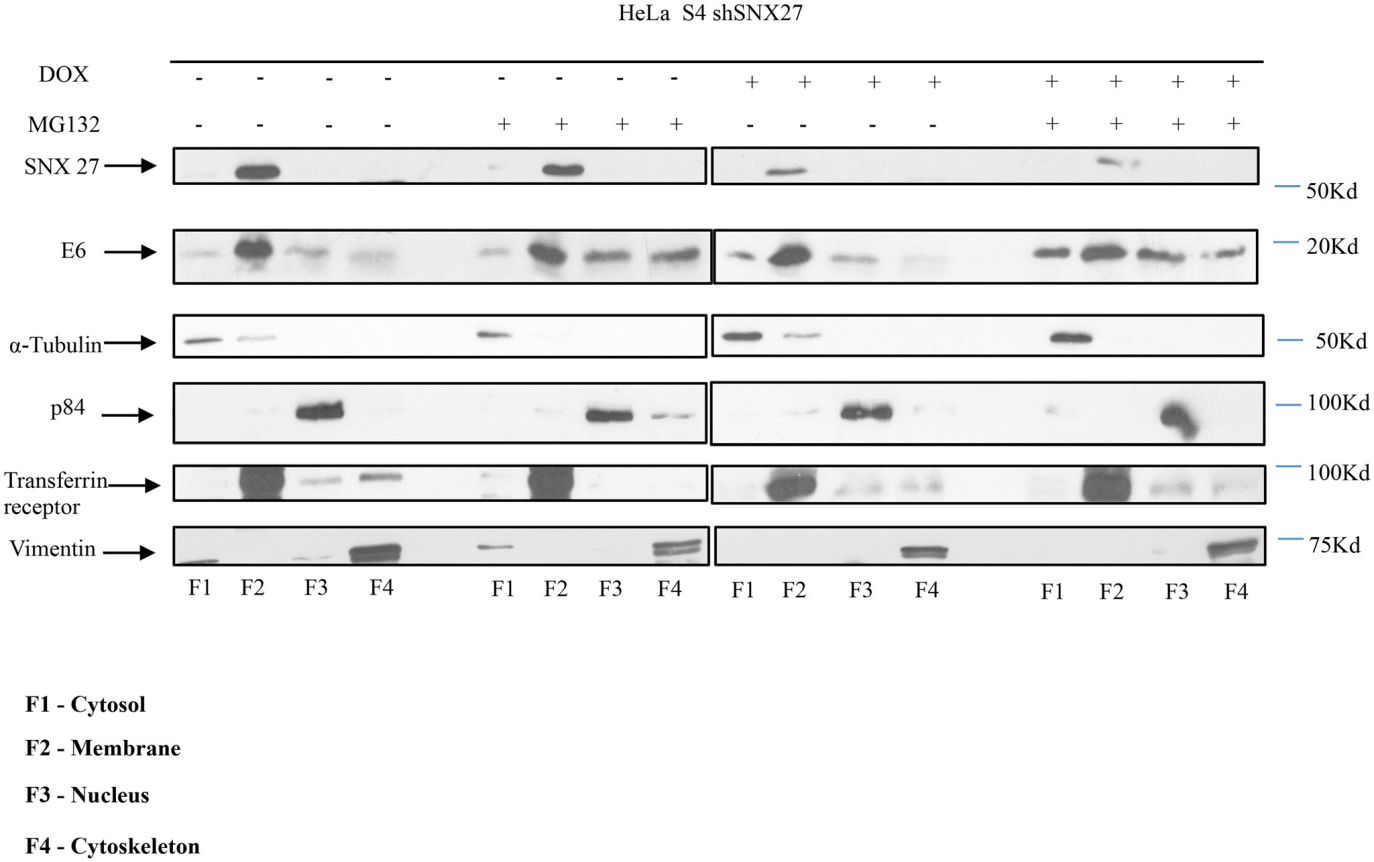
HPV-18 E6 resides primarily within the membrane fraction of HeLa cells. The stably transfected HeLa S4 shSNX27 cells were treated with Doxycycline (DOX) or DMSO for 72 hours. The cells were then treated with the proteosome inhibitor MG132 or control DMSO, as indicated, for a further 3 hours. The cells were then harvested and cytoplasmic, membrane, nuclear and cytoskeletal fractions were prepared using the ProteoExtract Cell Fractionation Kit (Calbiochem). The fractions were run on SDS polyacrylamide gels and HPV-18 E6 and SNX27 detected by western blotting. The arrows indicate the position of SNX27, HPV-18 E6, and the markers of the different fractions: α-Tubulin (cytoplasmic), Transferrin Receptor (membrane), p84 (nuclear) and Vimentin (cytoskeletal).

Many E6 interacting partners are targeted for ubiquitin-dependent proteasomal degradation. However, in an extensive series of in vitro and in vivo assays, we found no evidence that SNX27 was a degradation substrate for either HPV-16 or HPV-18 E6. Previous studies have reported E6 to be largely expressed in the nucleus, whilst SNX27 exists primarily within the early endocytic compartments in the cytoplasmic and membrane compartments of the cell (35). We were therefore first interested in determining whether HPV-18 E6 and SNX27 were present within similar cellular compartments. To do this we performed cell fractionations of HPV-18 positive HeLa cells, in the presence and absence of the proteasome inhibitor MG132 to rescue any pools of the two proteins that might be subject to proteasome-mediated degradation. In this analysis we also made use of a DOX-inducible shRNA to ablate SNX27 expression, to ascertain whether SNX27 might have any effect on the pattern of E6 expression. The results obtained are shown in Fig 3. As expected, the bulk of SNX27 is found within the membrane fractions of the cell (35). Most interestingly, the bulk of HPV-18 E6 in HeLa cells is also localised within the membrane compartment, suggesting a potentially similar location to SNX27, although loss of SNX27 has no major effect on the subcellular distribution of E6. In addition, the membrane-bound pool of E6 appears to be unaffected by proteasome inhibition, whilst there is a clear evidence of proteasome mediated regulation of E6 levels in the cytoplasmic, nuclear and cytoskeletal fractions.

**Fig 4:**
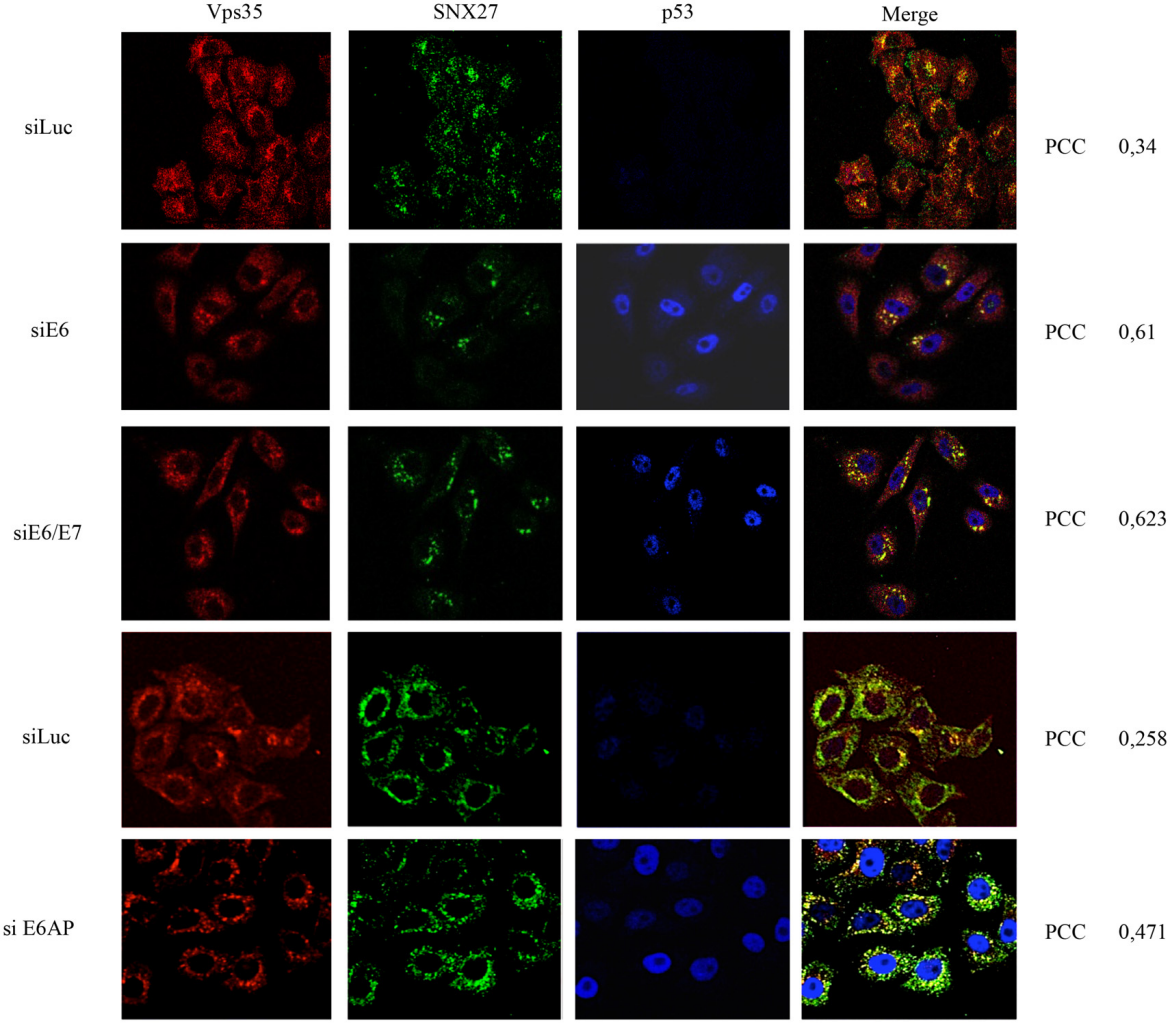
SNX27 accumulates in close proximity to retromer components in the absence of E6. HeLa cells were transfected with siRNA against Luciferase (siLuc), HPV-18 E6 (siE6), HPV-18 E6/E7 (siE6/E7) or E6AP (siE6AP) for 72 hours and the cells were then fixed using 4% Paraformaldehyde followed by permeabilization with PBS containing 0.1% Triton X-100. The cells were then stained for Vps35 (red) SNX27 (green) and p53 (blue) by indirect immunofluorescence. The images were acquired using the LSM 510 META Confocal Microscope (Carl Zeiss) and co-localisation between Vps35 and SNX27 was quantified by Velocity Software, with PCC values shown at the right hand side. Note the marked increase in co-localisation between SNX27 and Vps35 (given as Pearson’s Correlation Coefficient (PCC)) following ablation of E6, E6/E7 and E6AP.

Having found that E6 and SNX27 exist within the membrane fraction of HeLa cells, we were then interested in determining whether E6 could in any way modulate the subcellular localisation of SNX27. To do this, we analysed the distribution of SNX27 in HPV-18 positive HeLa cells, in the presence and absence of HPV-18 E6. The cells were transfected with control siRNA against Luciferase and siRNA against HPV-18 E6/E7 and E6AP as an alternative means of reducing E6 levels of expression [45]. After 72h, the cells were fixed and immunofluorescence analyses performed to detect SNX27, and also p53 as a control for the silencing of E6/E7 expression. We also analysed the distribution of the retromer component Vps35. The results in Fig 4 show a diffused speckled pattern of SNX27 distribution in control transfected cells, in agreement with previous studies (35), but a marked perinuclear accumulation, with increased levels of co-localisation with Vps35 following silencing of E6/E7 or E6AP, where a strong nuclear p53 staining can also be observed.

Taken together, these results suggest that E6 contributes to the maintenance of a dispersed pattern of Vps35 and SNX27 expression in HeLa cells, and that its loss results in a significant increase in Vps35/SNX27 co-localization at sites close to the nuclear membrane.

**Fig 5:**
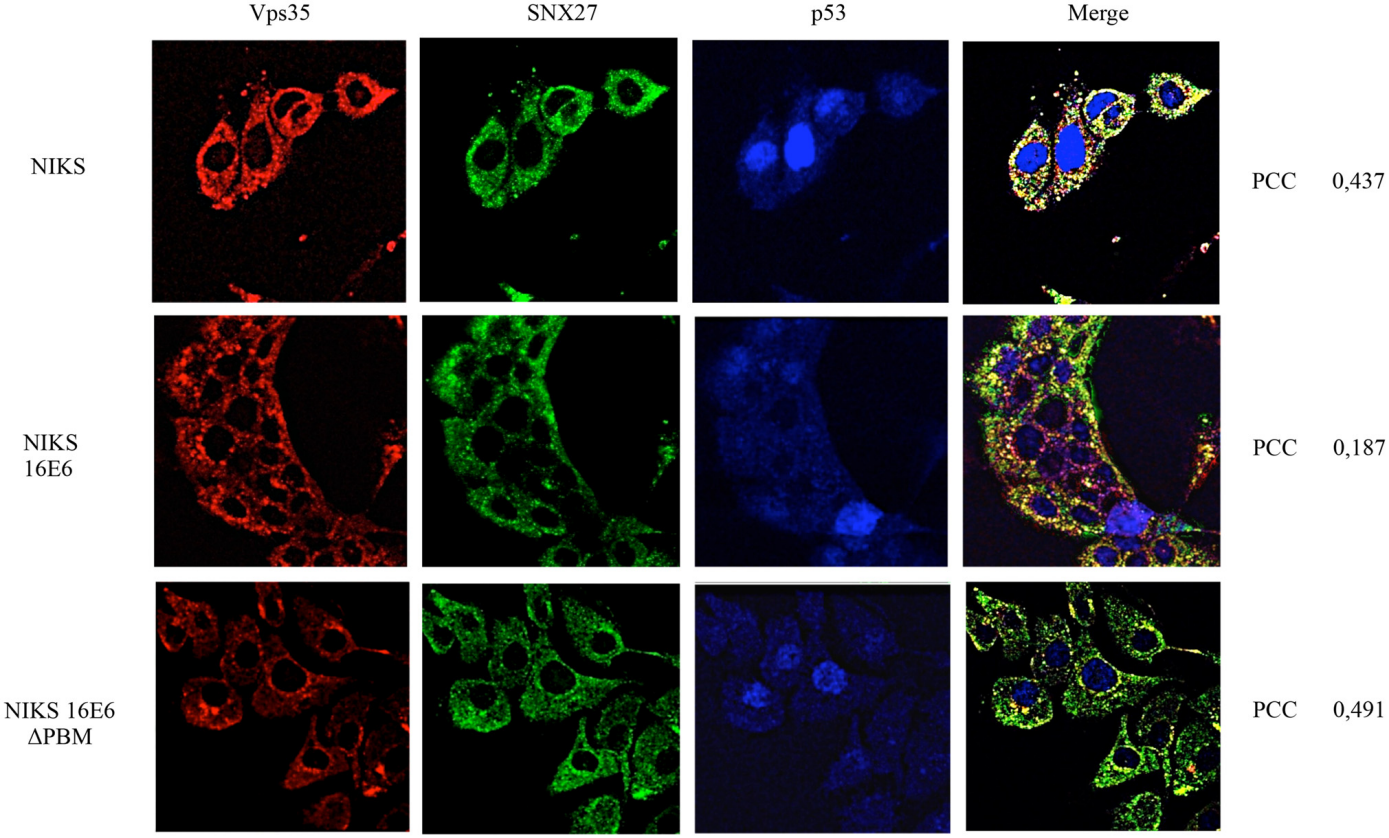
HPV-16 E6 modulation of SNX27 localisation is PBM-dependent. Control NIKS, NIKS expressing HPV-16 E6 and NIKS expressing the HPV-16 E6 ΔPBM mutant were fixed using 4% Paraformaldehyde followed by permeabilization with PBS containing 0.1% Triton X-100. The cells were then stained for Vps35 (red), SNX27 (green) and p53 (red) by indirect immunofluorescence. The images were acquired using the LSM 510 META Confocal Microscope (Carl Zeiss) and co-localisation between Vps35 and SNX27 quantified by Velocity Software, with PCC values shown at the right hand side. Note the marked increase in co-localisation between SNX27 and Vps35 in the control NIKS and NIKS containing E6ΔPBM.

To determine whether this change in the distribution of SNX27 is related to the ability of E6 to interact with SNX27, we analysed the distribution of SNX27 in the immortalised keratinocyte cell line NIKS, expressing either wild type HPV-16 E6 or a HPV-16 E6ΔPBM mutant. The cells were fixed and stained for SNX27 and Vps35 as described above. The results obtained in Fig 5 show significant co-localisation of Vps35 and SNX27 in the control NIKS not expressing E6, whilst this becomes much more dispersed in the wild type HPV-16 E6 expressing cells with limited areas of co-localisation. In contrast, the pattern of SNX27 and Vps35 distribution in the HPV-16 E6ΔPBM cells, shows increased levels of co-localisation of SNX27 and Vps35, reflecting more the pattern seen in the control NIKS. These results indicate that changes in the subcellular distribution of SNX27 and Vps35 in the presence of HPV E6 are in part dependent upon on intact E6 PBM.

### SNX27 and the retromer form a complex with E6 in HeLa cells

**Fig 6:**
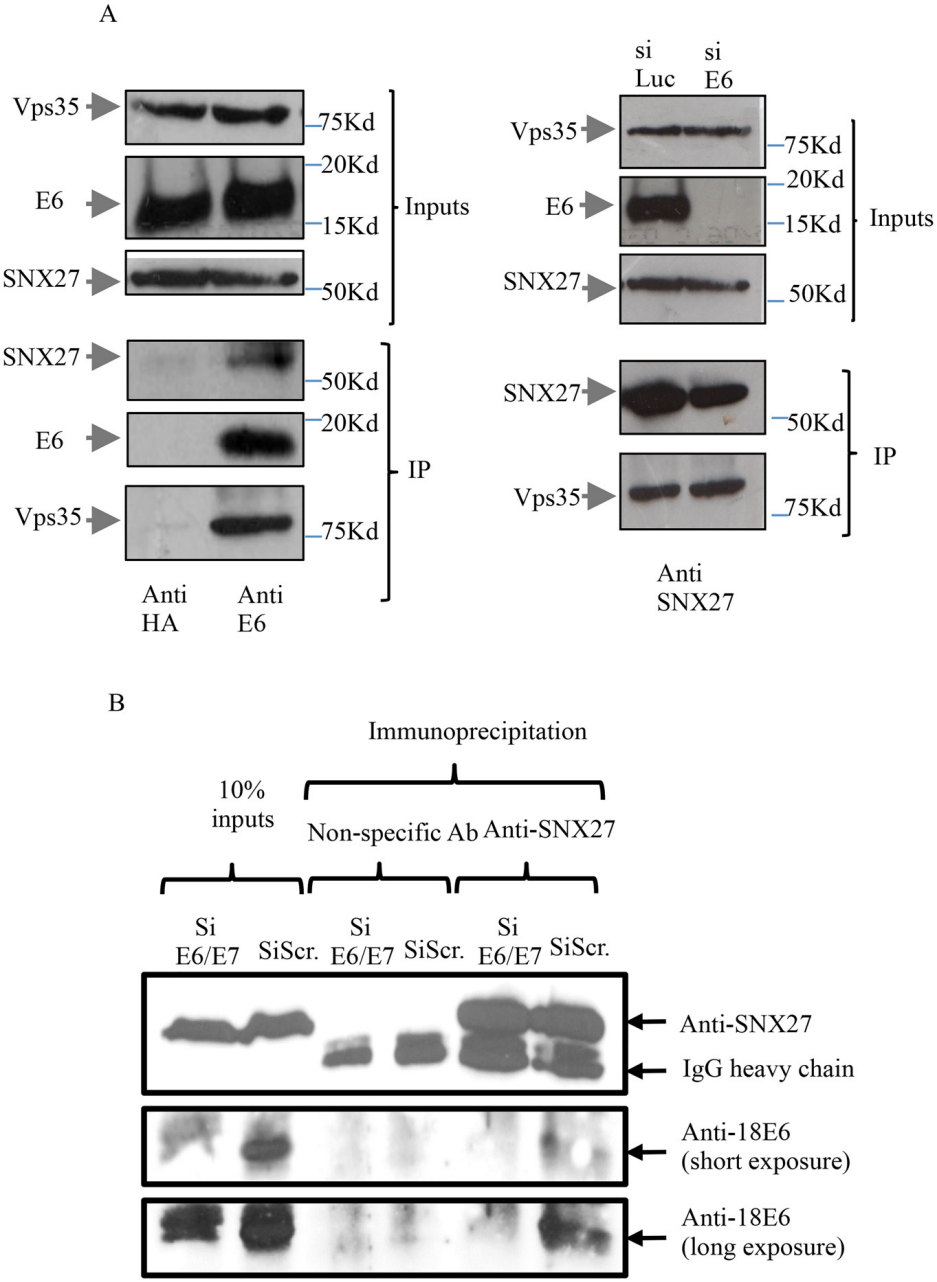
HPV-18 E6 exists in a complex with SNX27 and the retromer in HeLa cells. Panel A Left. HeLa cell extracts were incubated with anti-HPV-18 E6 monoclonal antibody or anti-HA monoclonal antibody as a negative control and the immune complexes were precipitated using Protein A Sepharose beads. The beads were washed and then loaded onto SDS polyacrylamide gels and co-immunoprecipitating proteins detected by SDS-PAGE followed by western blotting. The arrows indicate the position of SNX27, E6 and Vps35, with the upper three panels showing the levels of input proteins and the lower panels showing the immunoprecipitates. Panel A Right. HeLa cells were treated with siRNA against Luciferase as control or against HPV-18 E6 for 72 hours. Membrane fractions were prepared using the ProteoExtract Cell Fractionation Kit (Calbiochem) and then incubated with anti-SNX27 antibody and immune complexes were precipitated using Protein A Sepharose beads. The beads were washed, loaded onto SDS polyacrylamide gels and immunoprecipitating proteins detected by western blotting. The arrows indicate the position of SNX27 andVps35, with the upper two panels showing protein inputs and the two lower panels showing the immunoprecipitation. Panel B. HeLa cells were transfected with siE6/E7 or scrambled siRNA (siScr) and after 72hrs cells were harvested and immunoprecipitated using non-specific antibody or anti-SNX27. Complexes were then analysed by western blotting for HPV-18 E6 (anti-18 E6) and SNX27. Arrows indicate the positions of SNX27 and E6 together with the IgG heavy chain and a short and long exposure of the anti-18E6 western blot is provided for further clarity. Input proteins are also shown.

The above results indicate that E6 expression can alter the subcellular distribution of SNX27, and can affect the degree of its co-localisation with components of the retromer. We next wanted to determine whether E6 could modulate the degree of interaction between SNX27 and Vps35, and, furthermore, whether E6 could also be found in the SNX27/Vps35 complex in vivo. To do this, co-immunoprecipitation assays were performed where endogenous E6 from HeLa cells was immunoprecipitated using an anti-HPV-18 E6 monoclonal antibody. The immune complexes were then adsorbed on Protein A Sepharose beads and analyzed by SDS-PAGE followed by Western blotting for E6, SNX27 and Vps35. As can be seen from Fig 6A (left hand panel), both SNX27 and the Vps35 component of the retromer co-immunoprecipitate with E6, indicating that endogenous E6 is found in close proximity with the retromer. Likewise, HPV-18 E6 also co-immunoprecipitates with SNX27 when anti-SNX27 antibodies are used, and the specificity of this interaction is further demonstrated by loss of signal when the cells are transfected with siRNA against E6/E7 (Fig 6B). In order to determine whether E6 can affect association between SNX27 and Vps35, we performed an SNX27 immunoprecipitation from HeLa cells, which had been previously transfected with control siRNA luciferase or siRNA E6 to ablate E6 expression. The results in Fig 6A (right hand panel) show no major difference in the levels of SNX27/Vps35 interaction in the presence or absence of HPV-18 E6.

### Ablation of E6 expression leads to a reduction in GLUT-1 levels

**Fig 7:**
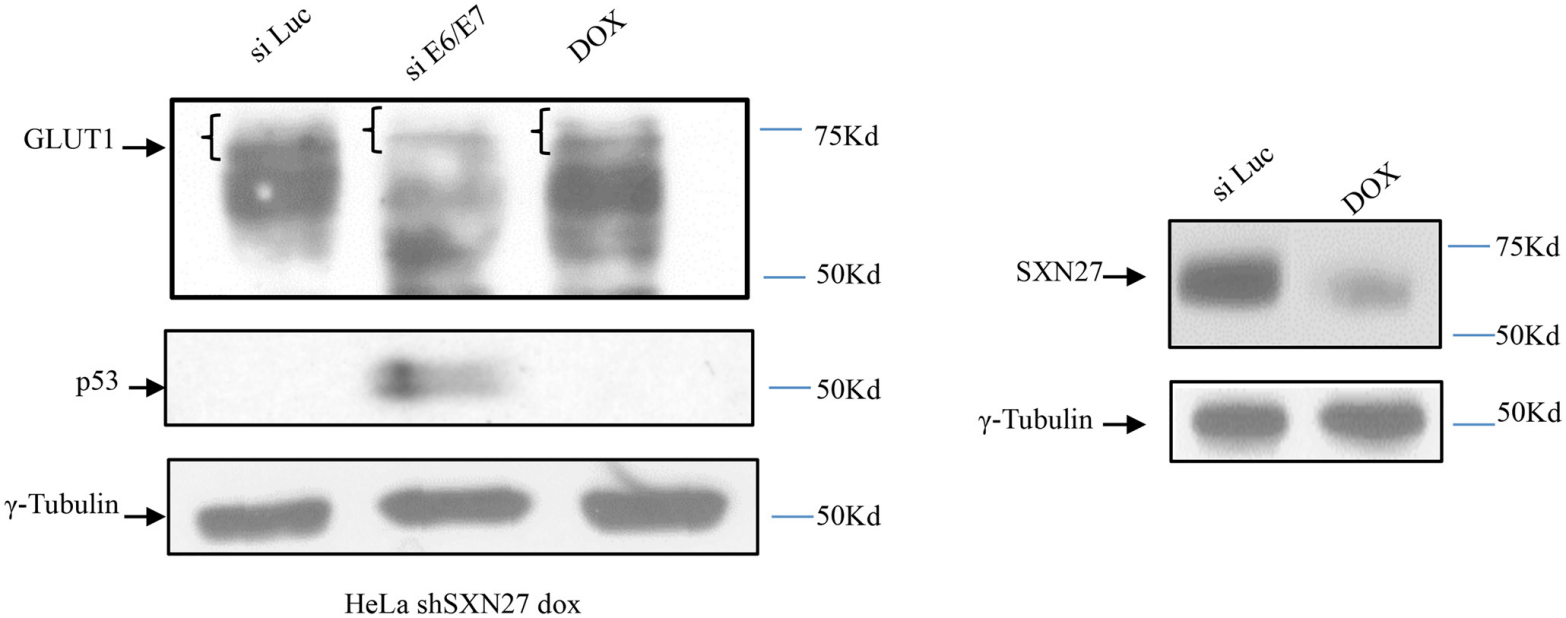
Loss of HPV-18 E6 alters GLUT-1 levels in HeLa cells. HeLa S4 cells were treated with siRNA against Luciferase, HPV-18 E6/E7 or DOX to induce SNX27 shRNA for 72 hours. The cells were then harvested and levels of GLUT-1 expression analysed by western blotting. Slower migrating forms of GLUT-1 are indicated by the brackets. Knock down of E6 and SNX27 were verified by western blotting for p53 and SNX27 respectively. The lower panel shows γ-tubulin as the loading control.

Previous studies have shown that the glucose transporter GLUT-1 requires both SNX27 and Vps35 for its efficient recycling back to the cell surface, with loss of SNX27 resulting in a marked decrease in the levels of GLUT-1 expression owing to its enhanced lysozomal degradation [40]. Since we observed that loss of E6 leads to alterations in the distribution of SNX27 and Vps35, we wanted to determine whether this might be reflected in changes in expression of a well-defined SNX27 cargo. To investigate this, we performed a series of assays to directly monitor the levels of expression of GLUT-1 in HeLa cells following ablation of either HPV-18 E6 or SNX27 expression. Cells were transfected with siRNA against Luciferase or siRNA against E6, or were treated with DOX to induce the SNX27 shRNA for 72h. After this time the cells were harvested and the levels of GLUT-1 expression analysed by western blotting. As a control for E6 ablation we monitored p53, and SNX27 was also analysed to ensure efficacy of the inducible shRNA. The results in Fig 7 demonstrate that GLUT-1 migrates as multiple bands, which is in agreement with previous studies. Most importantly however, there is a clear reduction in the levels of expression of the higher molecular weight, functional forms of the protein [46, 47], following loss of E6 or SNX27. These results demonstrate that removal of E6 in HeLa cells has a very similar effect upon GLUT-1 expression levels as removal of SNX27.

### The loss of E6 leads to an altered distribution of SNX27 cargo within endocytic compartments

**Fig 8:**
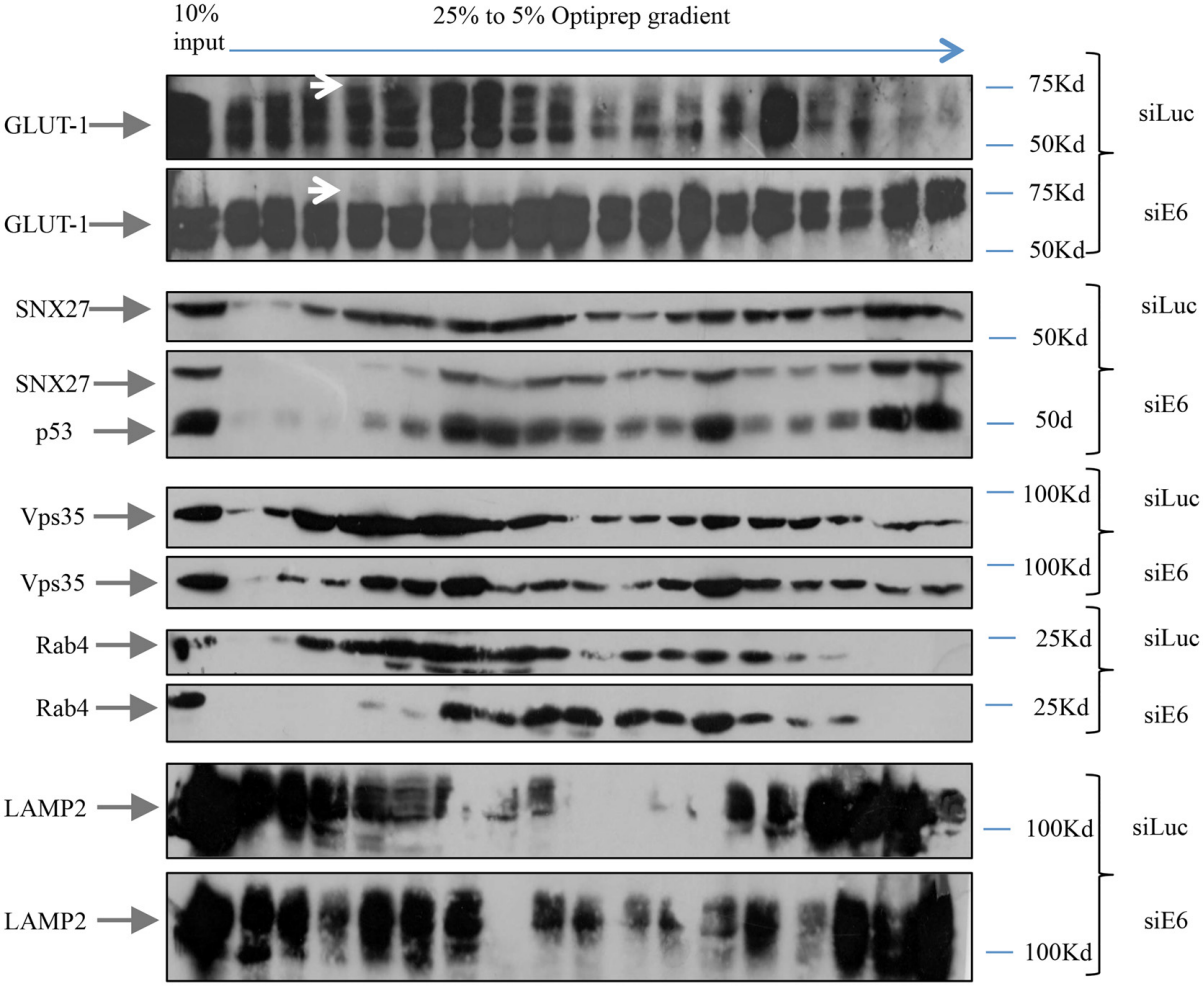
The loss of HPV-18 E6 in HeLa cells leads to an altered distribution of SNX27 and GLUT-1 cargo within endocytic compartments. HeLa cells were treated with siRNA against Luciferase or E6 for 72 hours and post nuclear extracts were loaded onto 5–25% OptiPrep gradients. The collected fractions were loaded on 12% SDS polyacrylamide gels followed by Western Blotting. The arrows indicate the position of GLUT-1, SNX27, p53 as a control for E6 knock down, but which was undetectable in the siLuc transfected cells, Rab4, Vps35 and LAMP2. The upper white arrows indicates the higher molecular weight forms of GLUT-1 that are absent following the loss of E6.

The above results demonstrate that GLUT-1 levels are reduced in HeLa cells lacking either E6 or SNX27. We were therefore interested in determining whether E6 had any influence on the composition of the endocytic compartments and their associated cargoes. To do this HeLa cells were transfected with siRNA against E6 or Luciferase for 72h and cell extracts subsequently fractionated on 5–25% OptiPrep gradients as described previously [48]. The fractions were then collected and analyzed by Western blotting. As can be seen in Fig 8, there are marked changes both in the distribution of cargoes and in the endocytic compartments following E6 ablation. In control transfected cells, the early endosome marker Rab4 appears to follow very closely the distribution of both SNX27 and Vps35. However, upon loss of E6 there is a clear shift in the distribution of both Rab4 and SNX27 within the gradient, whilst Vps35 appears to be relatively unchanged. Likewise, there is a corresponding marked shift in the distribution of the SNX27 cargo GLUT-1, and there is also an overall reduction in the levels of expression of the higher molecular weight forms of GLUT-1, similar to that seen in Fig 7. Interestingly there is no significant alteration in the mobility of the late endosomal marker LAMP2. These results demonstrate that in HeLa cells loss of E6 has quite a profound effect on the distribution of SNX27 within the cargo transporting apparatus, in support of the notion that E6 can modulate SNX27 function.

### E6 ablation leads to increased levels of cytoplasmic GLUT-1

**Fig 9:**
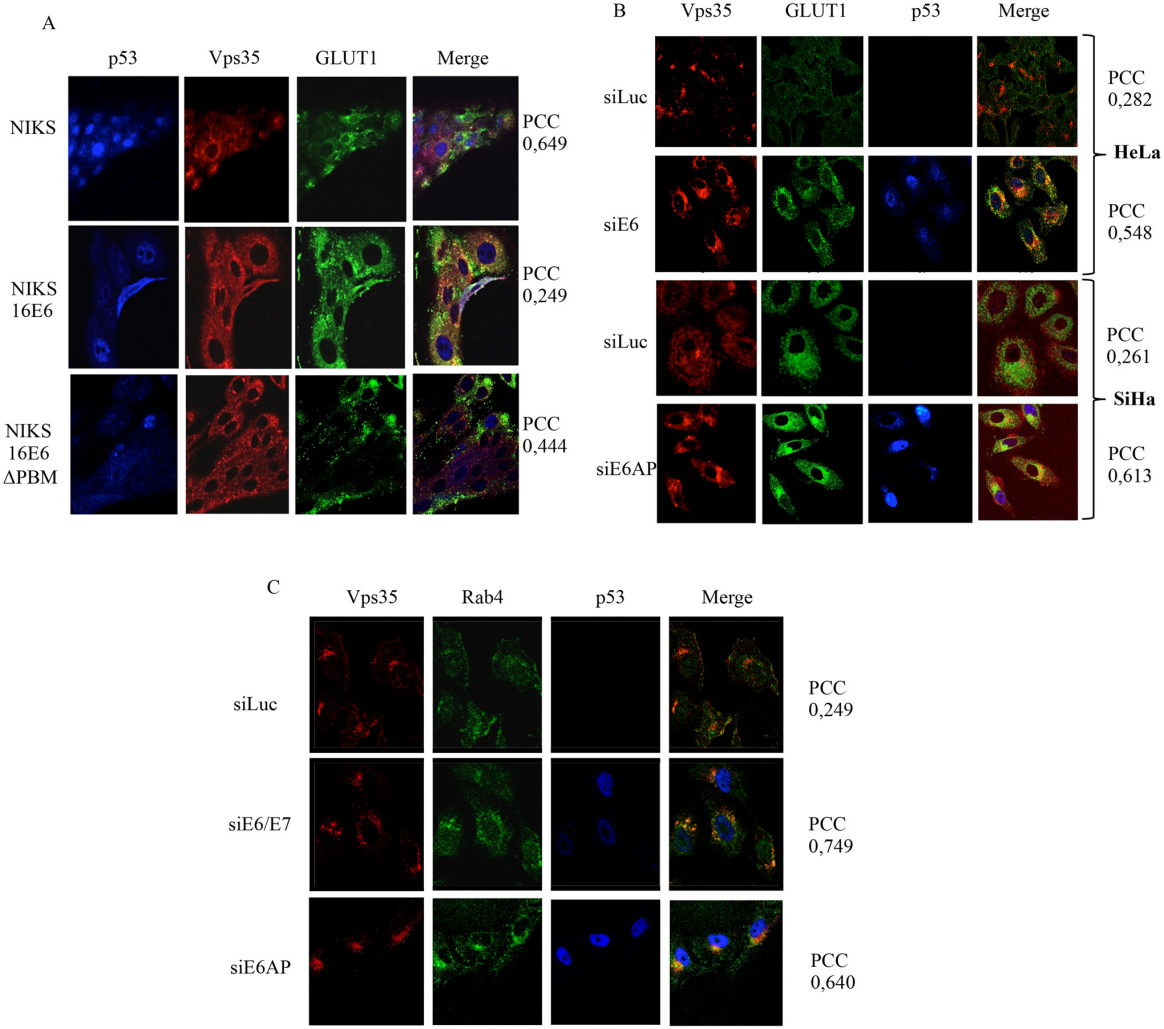
GLUT-1 distribution is modulated by E6 in a PBM-dependent manner. Panel A. Control NIKS, NIKS expressing HPV-16 E6 and NIKS expressing the HPV-16 E6ΔPBM mutant were fixed using 4% paraformaldehyde followed by permeabilization with PBS containing 0.1% Triton X-100. The cells were then stained for p53 (blue), Vps35 (red) and GLUT-1 (green) by indirect immunofluorescence. Co-localisation between Vps35 and GLUT-1 was measured using Velocity software and POC values are shown at the right. Panel B: HeLa and SiHa cells were transfected with siRNA against Luciferase, HPV-18 E6 or E6AP as indicated for 72 hours and the cells were then fixed using 4% paraformaldehyde followed by permeabilization with PBS containing 0.1% Triton X-100. The cells were then stained for Vps35 (red), GLUT-1 (green) and p53 (blue) by indirect immunofluorescence. The images were acquired using the LSM 510 META Confocal Microscope (Carl Zeiss). Co-localisation between GLUT1 and Vps35 was measured using Velocity software and PCC values are shown at the right hand side. Panel C: HeLa cells were transfected with siRNA against Luciferase or HPV-18 E6/E7 or E6AP for 72 hours and the cells were then fixed using 4% Paraformaldehyde followed by permeabilization with PBS containing 0.1% Triton X-100. The cells were then stained for Vps35 (red), Rab4 (green) and p53 (blue) by indirect immunofluorescence. The images were acquired using the LSM 510 META Confocal Microscope (Carl Zeiss). Co-localisation between GLUT-1 and Rab4 was measured using Velocity software and PCC values are shown at the right hand side.

The above data suggest a significant shift in the distribution of several endocytic compartments and of at least one associated cargo, GLUT-1, following the ablation of E6 expression in HeLa cells. We were therefore interested in determining whether this alteration in the pattern of GLUT-1 expression could be visualised by immunofluorescence analysis. Cells were transfected with siRNA against Luciferase or E6 and, after 72h, analysed by immunofluorescence for GLUT-1 and Vps35 localisation, with p53 serving as a positive control for E6 ablation. As can be seen from Fig 9B, most of the GLUT-1 appears to be localized on the cell periphery in the control cells, while the Vps35 remains distributed throughout the cytoplasm as seen before. In contrast, upon E6 ablation, the majority of GLUT-1 is located in intracellular vesicle-like structures with a pool showing significant co-localization with Vps35. Similar results are also obtained in SiHa cells following ablation of E6AP expression, which we have shown previously to result in a loss of E6 [45]. In addition, analysis of GLUT-1 distribution within NIKS cells (Fig 9A) also shows a clear E6 PBM-dependent effect upon the pattern of GLUT-1 expression, although the specific distribution varies between the different cell types analysed. Taken together, these results indicate that the loss of E6 leads to an increased cytoplasmic expression of GLUT-1, a subset of which accumulates in close proximity to a component of the retromer complex.

The results in Fig 8 indicated a change in the localisation of the early endosomal marker Rab4. We therefore proceeded to investigate whether this was also reflected in a change in its subcellular distribution following ablation of E6 expression. The results in Fig 9C indeed show a significant alteration in the pattern of Rab4 distribution in HeLa cells following ablation of either E6 or E6AP expression. Once again there appears to be increased accumulation in close proximity to the Vps35 component of the retromer.

### Loss of HPV-18 E6 causes a reduction in glucose uptake in HeLa cells

**Fig 10:**
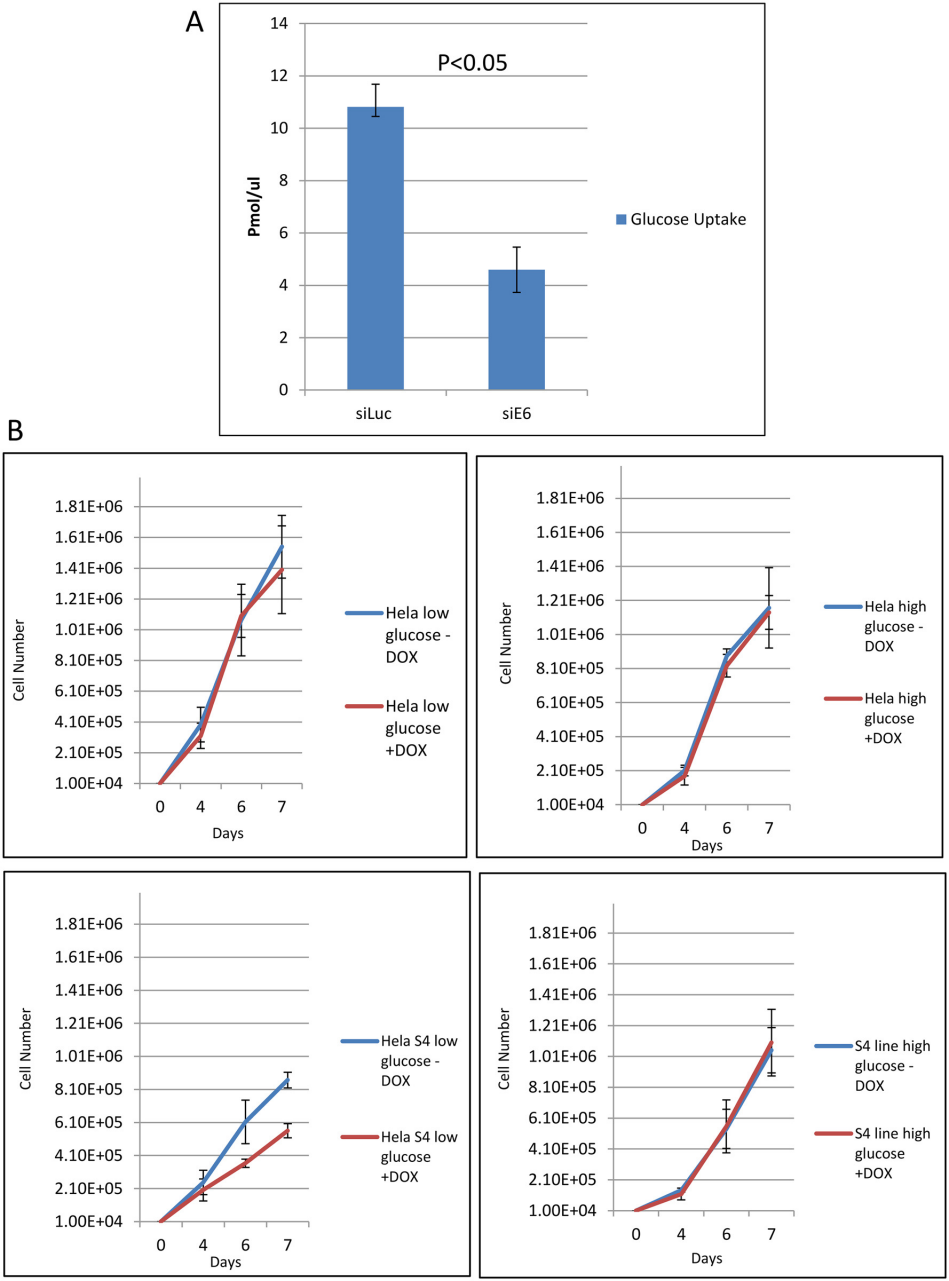
Modulation of glucose uptake and cell proliferation by E6 and SNX27. Panel A. HeLa cells were treated with siRNA against Luciferase or HPV-18 E6 for 72 hours and glucose uptake was analyzed using the glucose analog 2-deoxyglucose (2-DG) using the Colorimetric Glucose Uptake Assay Kit (Abcam) according to the manufacturer’s instructions. The graph shows the cumulative results of three independent experiments, with 2-DG uptake measured in pmol/μl. Error bars represent standard deviations with a p-value < 0.05. Panel B. Control HeLa cells and HeLa S4 DOX inducible shRNA SNX27 cells were plated in medium containing low or high glucose in the presence and absence of DOX as indicated. Cells were then counted over a period of 4 days and the numbers represent the means from three independent assays. Standard deviations are shown.

The above data demonstrate that E6 can modulate the pattern of SNX27 association with the endosomal transport machinery in a PBM-dependent manner. A consequence of this appears to be reflected in changes to GLUT-1 association with endosomal recycling complexes, with loss of E6 resulting in lower levels of GLUT-1 expression and altered intracellular distribution. Therefore we next wanted to determine whether this alteration in GLUT-1 transport was also reflected in an alteration of its function as a glucose transporter. To do this, HeLa cells were treated with siRNA against E6, or Luciferase as control, for 72h and glucose uptake was then measured using the analog 2-deoxyglucose (2-DG). As can be seen from Fig 10A, there is a significant decrease in the amount of glucose that is taken up by HeLa cells when E6 is depleted, compared with the Luciferase control. These data show that the depletion of GLUT-1upon E6 ablation is reflected in a reduction in glucose uptake by these cells, and reflects reports of similar results seeing loss of GLUT-1 expression following SNX27 knockdown [40]. This demonstrates a direct functional consequence for HPV-18 E6 modulation of the SNX27-retromer cargo transport pathway.

The above results indicate that SNX27 plays an important role in glucose uptake, an activity that is modulated by E6. They also suggest that under conditions of low glucose availability, loss of SNX27 might result in reduced rates of cell proliferation. To investigate this possibility, control HeLa cells and shRNA SNX27 HeLa cells were plated in low and high glucose, both in the absence and presence of DOX to induce the SNX27 shRNA. The cells were then counted over a period of days and the results obtained are shown in Fig 10B. As can be seen, loss of SNX27 in conditions of low glucose has a marked inhibitory effect upon continued cell proliferation, whilst high glucose availability largely mitigates these effects. These results confirm that SNX27 contributes directly towards nutrient uptake in HPV-18 positive HeLa cells.

## Discussion

In this study we have identified a novel activity of the high risk HPV E6 oncoproteins, linking them to the modulation of endosomal transport pathways. This appears to be mediated, at least in part, through a direct interaction between the high risk HPV E6 oncoproteins and the cellular SNX27, a protein that controls cargo fate determination in endocytic recycling. One consequence of this interaction is modulation of the endocytic transport of the glucose transporter GLUT-1, which subsequently affects the amount of glucose uptake in HPV-positive tumour cells. These results suggest that E6 can directly affect the nutrient balance in HPV infected cells, through modulation of endocytic recycling pathways to maintain sufficient nutrients for cell survival during the HPV life cycle and in progression to malignancy.

Previous proteomic analyses had suggested that HPV-18 E6 could potentially interact with different components of the endocytic sorting machinery, including SNX27 and the retromer components Vps35 and Vps26, indicating that E6 might be in close proximity to the retromer complex [23,24]. To investigate this in more detail we first performed a series of in vitro interaction assays and found that multiple high risk HPV E6 oncoproteins all share a similar ability to interact with SNX27. Mutational analyses demonstrated that the principal mode of recognition was through classic PBM-PDZ recognition, although there were some subtle differences from other reported E6 interactions with PDZ domain-containing substrates. In particular, the number of residues in the region of the E6 PBM that were critically required for SNX27 PDZ recognition appeared to be fewer than those required for MAGI or Dlg recognition. In addition, ablation of the E6 PBM or the core region of the SNX27 PDZ domain still allowed a low level of interaction, suggesting the existence of additional means by which E6 can interact with SNX27. In support of this, a weak but consistent interaction was also observed between HPV-11 E6 and SNX27, suggesting that modulation of endocytic transport might also be a feature of low risk HPV types, and this is currently a subject for further investigation.

In agreement with the proteomic analyses (23), we also found that HPV-18 E6 could co-immunoprecipitate both SNX27 and the Vps35 component of the retromer from cells, confirming a close association of E6 with the retromer complex. Indeed, cell fractionation studies demonstrated that the bulk of endogenously expressed E6 in HeLa cells is actually present within the membrane fraction of the cell, which is very similar to the localisation seen for SNX27. Throughout, we found no compelling evidence to suggest that E6 can influence the turnover of SNX27 through proteasome-dependent pathways. However, it was interesting to note that the membrane-bound forms of E6 were not subject to proteasome-mediated degradation, as there was no change in the levels of E6 expression in this fraction of the cell following proteasome inhibition. This contrasts with the situation with the cytoplasmic, nuclear and cytoskeletal pools, where all these forms of E6 were rescued following proteasome inhibition. It should also be mentioned that when E6 is overexpressed using exogenous transfection of expression plasmids, we invariably detect the bulk of E6 within the cytoskeletal compartment of the cell, emphasising the need to study the endogenously expressed E6 for these types of analyses.

Having defined SNX27 as a novel interacting partner of HPV-18 E6, we then proceeded to investigate the potential consequences of this interaction. Clearly, removal of E6 from HPV-18 positive HeLa cells has quite a marked effect on the subcellular distribution of SNX27, with significant accumulation in regions proximal to the nucleus and an apparent increase in the co-localisation with Vps35. To confirm that these alterations in the pattern of SNX27 expression were PBM-dependent, we also analysed the distribution of SNX27 in control NIKS, and in NIKS expressing HPV-16 E6 or HPV-16E6 ΔPBM. Again, there was significant co-localisation between SNX27 and Vps35 in the control NIKS and HPV-16 E6ΔPBM lines, but this was greatly decreased in the wild type E6-expressing cells. Taken together these studies suggest that the E6 association with SNX27 can modulate its subcellular distribution. To further investigate this we then analysed the distribution of SNX27 in different endosomal/lysosomal fractions using OptiPrep gradients to fractionate the subcellular membrane compartments in HeLa cells. Again, loss of E6 resulted in a marked shift in the distribution of SNX27 within the gradients. SNX27 remained in close proximity to the early endosomal marker Rab4, but shifted with respect to some of the Vps35 component of the retromer. Albeit less clearly, the GLUT-1 SNX27 cargo also appeared to shift in the gradient, suggesting that loss of E6 could also affect the distribution of an SNX27 cargo. These results were independently verified by immunofluorescence analyses, which also demonstrated an alteration in the co-localisation of Rab4 with Vps35, together with an increase in cytoplasmic GLUT-1 in cells where E6 expression had been ablated.

Having defined a role for the E6 PBM in modulating components of the endosomal transport machinery, we then proceeded to assess the effects on the function of GLUT-1. In both total cell extracts and in the gradient analyses, loss of E6 resulted in a marked decrease in the levels of expression of the functional higher molecular weight forms of GLUT-1. Similar effects were also observed following loss of SNX27 expression. Functionally, this loss of E6 resulted in a reduction in the levels of glucose uptake, suggesting that a direct consequence of E6 modulation of SNX27 function is an increase in the rates of glucose uptake, thereby favouring a more efficient use of available nutrients for continued cell proliferation and cell survival. In order to confirm that SNX27 does indeed play a significant biological role in HeLa cells we also measured cell growth following ablation of SNX27 expression under different growth conditions, and clearly found a reduction in cell proliferation when cells were grown in a low glucose medium. Taken together these results demonstrate that the association between E6 and SNX27 can modulate the endocytic transport of the GLUT-1 glucose transporter, one consequence of which is maintenance of cell proliferation under low nutrient conditions.

Several major questions arise from these studies. The first relates to the precise mechanism by which E6 can modulate SNX27 function. The interaction is largely PBM-PDZ mediated, although not exclusively. It seems unlikely that E6 and GLUT-1 could occupy the SNX27 PDZ pocket simultaneously; however recent studies have shown that the GLUT-1 affinity for the SNX27 PDZ domain is significantly higher than that of E6 [49]. This suggests that E6 association might be transitory, and be replaced in the PDZ pocket by high affinity cargoes. It remains to be determined whether the weaker association of E6 with SNX27 might also then contribute to a modulation of the sorting process, or whether the E6 PBM helps recruit SNX27 to certain endocytic compartments, favouring faster recycling. It is also possible that other, more weakly bound, cargoes of SNX27 might actually be out-competed for binding SNX27 by E6, thereby inhibiting their recycling.

Recent studies have shown an important role for the retromer in the function of SNX27, and certainly E6 appears to exist in a complex with SNX27 bound to Vps35, without apparently affecting the biochemical levels of either SNX27 or Vps35 [40,44]. Nonetheless depletion of E6 does seem to affect the degree of co-localisation of SNX27 with Vps35, both in immunofluorescence experiments and in gradient fractionations. In one of these assays, this modulation is shown to be PBM dependent. This suggests that E6 can modulate the endocytic compartment to which SNX27 is recruited, suggesting that the time spent in close proximity with Vps35 is reduced in the presence of E6. Dissecting the molecular basis for this will be an important avenue for future investigation.

Taken together, these studies indicate the existence of a novel activity for the HPV E6 oncoproteins, linking them directly to the modulation of endosomal transport pathways, and suggesting a completely novel way of modulating the cellular homeostasis, both during viral infection and in the development of malignancy.

## Materials and Methods

### Cell culture and transfections

HeLa (ATCC) and HeLa S4 shSNX27 (38) and SiHa (ATCC) cells were maintained in Dulbecco’s Modified Eagles’s Medium (DMEM) supplemented with 10% Fetal Calf Serum (Life Technology), penicillin-streptomycin (100 U/ml) and glutamine (300 μg/ml). Cells were cultured at 37°C with 10% CO_2_. The NIKS (Normal Immortalised Keratinocytes [50]) control, NIKS 16 E6 and NIKS 16 E6ΔPBM [51] cells were maintained in F medium (0.66 mM Ca^2+^) composed of 3 parts Ham's F12 medium to 1 part Dulbecco's modified Eagle's medium and supplemented with the following components: 5% fetal bovine serum (FBS), adenine (24 μg/ml), cholera toxin (8.4 ng/ml), epidermal growth factor (10 ng/ml), hydrocortisone (2.4 μg/ml), and insulin (5 μg/ml).

HeLa cells were transfected with siRNA using Lipofectamine RNAiMAX transfection reagent (Invitrogen). HeLa S4 shSNX27 cells were treated with 0.2 mg/ml Doxycycline to induce the shRNA. The following siRNAs were used: HPV-18 E6 and HPV-18 E6/E7 were custom synthesised by Dharmacon whilst the E6AP and SNX27 siRNAs were a Dharmacon Smart Pools.

### Plasmids

GST fusion proteins were generated from pGEX2T plasmids expressing HPV-11 E6, 16 E6, 18 E6, 31 E6, 33 E6, 51 E6 and 58 E6 proteins, SNX27 [37], HPV-18 E6ΔPBM and HPV-18 E6 T156E as described previously [52]. The SNX27 deletion mutants (Δ67–77, Δ97–110 and Δ113–121) were prepared using primers against the specified regions using the pCI Neo Myc tagged SNX27 as template. The HPV-18 E6 C-terminal mutants were used for the *in vitro* binding assays as described previously [41]. All expression constructs were transformed into *E*.*coli* strain DH5α.

### In vitro binding assays

Purified GST fusion proteins were incubated with *in vitro* translated and radiolabelled proteins as indicated for 2 hours at room temperature. Proteins were translated *in vitro* using a Promega TNT Rabbit Reticulocyte Lystate kit and radiolabelled with [S^35^] Cysteine or [S^35^] Methionine (Perkin Elmer). Equal amounts of translated proteins were mixed with the GST fusion proteins immobilized on glutathione agarose beads and incubated on a rotating wheel. The beads were then washed thrice with PBS containing 1% Triton X-100 and analyzed by SDS-PAGE followed by autoradiography.

### Co-immunoprecipitation assays

For endogenous protein co-immunoprecipitation assays, HeLa cells were seeded in 10 cm dishes and either left untreated or treated with siRNA against Luciferase or E6 as indicated for 72 hours. Cell lysates were prepared either in Lysis Buffer (20mM Tris pH 7.5, 150mM NaCl, 1mM EDTA, 1mM EGTA, 1% Triton X-100) containing protease inhibitors (Calbiochem Protease Cocktail 1); or they were extracted using the ProteoExtract Cell Fractionation Kit (Calbiochem). Then extracts were incubated with either anti-HA antibody (Roche), anti 18E6 antibody (ArborVita) or anti-SNX27 antibody (Abcam) as indicated overnight on a rotating wheel at 4°C. The immune complexes were captured using Protein A Sepharose beads and analyzed by SDS-PAGE followed by Western Blotting using anti-SNX27 antibody (Abcam), anti-Vps35 antibody (Abcam) and anti-18E6 antibody (ArborVita).

### siRNA and cell fractionation assays

For cell fractionation studies, HeLa S4 shSNX27 cells were seeded on 6cm dishes at a density of approximately 1.2 x 10^5^ and treated with Doxycycline or DMSO as indicated for 72 hours. The cells were then treated with MG132 or DMSO as indicated for 3 hours. Cells were collected by trypsinization and fractionated into cytoplasmic, membrane, nuclear and cytoskeletal fractions using the ProteoExtract Cell Fractionation Kit (Calbiochem) according to the manufacturer’s instructions and fractions were analyzed by SDS-PAGE followed by Western Blotting using anti-SNX27 antibody (Abcam), anti-18E6 antibody (ArborVita AVC#399), anti-α tubulin antibody (Sigma Aldrich), anti-p84 antibody (Abcam), anti-Transferrin Receptor antibody (Santa Cruz) or anti-Vimentin antibody (Santa Cruz) as indicated.

### Immunofluorescence assays

Cells were seeded on glass coverslips at a density of approximately 1.2 x 10^5^ cells and transfected with siRNA against Luciferase, E6 or E6/E7 as indicated for 72 hours. The cells were fixed using 4% Paraformaldehyde and permeabilized using PBS containing 0.1% Triton X-100. Immunostaining was performed by incubating the coverslips in PBS containing antibodies against SNX27 (Abcam), Vps35 (Abcam), GLUT-1 (Abcam), p53 (Santa Cruz) or Rab4 (Abcam) as indicated overnight in a humidified chamber at 4°C. The coverslips were then washed thrice with PBS and incubated with the respective fluorophore conjugated secondary antibodies as indicated for 1 hour in a humidified chamber at 37°C. The coverslips were then washed thrice with PBS and twice with distilled water and mounted onto glass slides. The images were captured using the LSM510 META Confocal Microscope (Carl Zeiss) and co-localisations were quantified using the Velocity Software and Pearson’s Correlation Coefficient (PCC) calculated for each set of images, where values closer to 1 indicate closer degrees of colocalisation, whilst a value of zero would indicate no co-localisation.

### Gradient assays

HeLa cells were seeded in 10cm dishes and transfected with siRNA against Luciferase or E6 for 72 hours. Optiprep gradients (5%-25%) were prepared as described previously [44] and equilibrated at room temperature for 3 hours. Cell extracts were prepared in the homogenization buffer containing protease inhibitors as described previously [44], and the lysates were syringed to ensure breakdown of the cells. The lysates were centrifuged at 3000 x g for 5 minutes at 4°C and the post nuclear extracts (supernatants) were loaded onto the equilibrated gradients. The gradients were centrifuged at 32,000 rpm for 18 hours at 4°C and fractions were collected using a peristaltic pump. The collected fractions were mixed with chilled acetone and incubated at -20°C overnight to precipitate the proteins. The precipitates were centrifuged at 14,000 rpm for 10 minutes at 4°C and washed once with chilled absolute ethanol. The precipitates were air dried and dissolved in 2x Laemmeli’s buffer. The fractions were loaded onto 12% SDS polyacrylamide gels and the endocytic profiles were analyzed by Western Blotting using anti-GLUT-1 antibody (Abcam), anti SNX27 antibody (Abcam), anti-p53 antibody (Santa Cruz), anti- Vps35 antibody (Abcam), anti- Rab4 antibody (Abcam) and anti- LAMP2 antibody (Abcam) as indicated.

### Glucose uptake assay

HeLa cells were seeded at a density of approximately 1.2 x 10^5^ cells in 6cm dishes and transfected with siRNA against Luciferase or E6 as indicated using the Lipofectamine RNAiMAX transfection reagent (Invitrogen) for 72 hours. Glucose uptake was measured using the glucose analog 2-Deoxyglucose (2-DG) which can be taken up by the cells but is not metabolized. The uptake of 2-DG was measured colorimetrically using the Glucose Uptake Assay Kit (Abcam) as per the manufacturer’s instructions. The glucose uptake is measured as picomoles/μl and the measurements from three independent assays were used to generate the graph and standard deviations.

### Cell proliferation assay

Control HeLa cells and HeLa cells (S4) containing DOX inducible SNX27 shRNA were plated in high (4.5g/l) or low glucose (1g/l) in the presence or absence of DOX to induce SNX27 knockdown. Cell numbers were then counted over a period of 4 days.
